# The sustainability of Lean in pediatric healthcare: a realist review

**DOI:** 10.1186/s13643-018-0800-z

**Published:** 2018-09-11

**Authors:** Rachel Flynn, Amanda S. Newton, Thomas Rotter, Dawn Hartfield, Sarah Walton, Michelle Fiander, Shannon D. Scott

**Affiliations:** 1 Faculty of Nursing, Level 3, Edmonton Clinic Health Academy, University of Alberta, 11405 87 Avenue, Edmonton, AB T6G 1C9 Canada; 2 Department of Pediatrics, Faculty of Medicine and Dentistry, University of Alberta, 11405 87 Avenue, Edmonton, AB T6G 1C9 Canada; 3 Healthcare Quality Programs, Queen’s University School of Nursing, Kingston, ON K7L 3N6 Canada; 4 Department of Pharmacotherapy, College of Pharmacy, University of Utah, L.S. Skaggs Building 4838, Salt Lake City, UT 84112 USA

**Keywords:** Realist review, Lean, Sustainability, Complex intervention, Quality improvement, Implementation science, Pediatric healthcare

## Abstract

**Background:**

Lean is a quality improvement management system from the Toyota manufacturing industry. Since the early 2000’s, Lean has been used as an intervention for healthcare improvement. Lean is intended to reduce costs and improve customer value through continuous improvement. Despite its extensive use, the contextual factors and mechanisms that influence the sustainability of Lean in healthcare have not been well studied. Realist synthesis is one approach to “unpack” the causal explanations of how and why Lean is sustained or not in healthcare. We conducted a realist synthesis using the context (C) + mechanim (M) = outcome (O) heuristic, to further develop and refine an initial program theory with seven CMO hypotheses, on the sustainability of Lean efforts across pediatric healthcare.

**Methods:**

Our search strategy was multi-pronged, iterative, and purposeful in nature, consisting of database, gray literature, and contact with three healthcare organizations known for Lean implementation. We included primary research studies, published and unpublished case studies or reports, if they included Lean implementation with a pediatric focus and sustainability outcome. We used the Normalization Process Theory and the National Health Services Sustainability Model, an operational definition for Lean and a comprehensive definition for sustainability as guidance for data extraction and analysis. Our initial program theory with was refined using a blend of abductive and retroductive analytical processes.

**Results:**

We identified six published primary research studies, two published quality improvement case studies, and three unpublished quality improvement case reports. Five CMO hypotheses from our initial program theory were substantially supported after synthesis, “sense-making and value congruency,” “staff engagement and empowerment,” and the “ripple effect” or causal pathway between Lean implementation outcomes that served as facilitating or hindering contexts for sustainability. Overall, there was variation with the conceptualization and measurement of sustainability.

**Conclusions:**

This study is the first to examine Lean sustainability in pediatric healthcare using realist methods. Future research should examine whether the predictors of implementation are the same or different to sustainability and evaluate the underlying mechanisms that influence the sustainability of Lean. There is also a need for research to develop and test conceptual models and frameworks on sustainability.

**Systematic review registration:**

PROSPERO-CRD42015032252.

**Electronic supplementary material:**

The online version of this article (10.1186/s13643-018-0800-z) contains supplementary material, which is available to authorized users.

## Background

The goal of Lean management systems is to reduce costs and increase value for customers through the creation of a continuous quality improvement (QI) culture [1, 2]. Lean originated from the Toyota automotive manufacturing industry in the 1930’s [1]. Toyota is one of the world’s most successful companies in the car manufacturing industry. In 2018 Toyota ranked number nine on Forbes list of the world's most valuable brands [3]. Given these presumptive positive outcomes of Lean management, it has become an attractive option for healthcare systems faced with demands to improve quality, increase efficiency, and decrease expenditure [4]. Internationally, Lean is increasingly applied to healthcare systems for improvement. Successful implementations of Lean in healthcare report waste reduction and increased efficiency [5–8]; while unsuccessful implementations have described Lean as inappropriate for healthcare and reported superficial adoption, system dysfunction, and disengaged staff [9–11].

Given the complexity of healthcare systems [12], contrary findings in the literature are not surprising. In addition to healthcare complexity, the extent of Lean implementation varies substantially [10, 13]. Virginia Mason, a private, non-profit medical center in Seattle, United States, for example, adopted Lean as a guiding philosophy across all departments and management systems—a macro level implementation, but most healthcare organizations adopt Lean at meso levels in efforts to improve a specific process or procedure. Seventy-three percent of Canadian health regions have indicated that Lean was a component of their organizational strategy [14], yet few regions have embraced it as their overarching approach to transform organizational culture and performance. The distinction between meso and macro adoption of Lean may be crucial to better understand the sustainability of Lean implementation efforts in healthcare.

Lean was not intended to be complex. It was intended to be a simple philosophy and management system for continuous improvement in the car manufacturing industry. The philosophy of Lean was to reduce waste, add value and create efficiency, through a set of activities and core principles. However, we argue in the context of healthcare, Lean is a complex intervention for improvement. There are a number of reviews on Lean in healthcare [5–7, 15] but none on the sustainability of Lean efforts or Lean in pediatric healthcare. Sustainability is a key implementation outcome, yet remains one of the least understood issues for implementation research [16]. Implementation of interventions for improvement is meaningless without including long-term sustainability efforts [17]. There are two defining characteristics of Lean: Lean philosophy and Lean activities. Lean philosophy is made up of two components: a commitment to Lean principles and a commitment to continuous improvement [18]. Lean implementation requires engagement of providers, followed by establishment and embedding of improvement behaviors [19].Considering these long-term aspects of Lean, evaluating sustainability is imperative.

In order to address the question of sustainability of Lean implementation in healthcare settings, it is necessary to understand the contextual factors and mechanisms that lead to outcomes. There is an argued case to shift from knowing whether a complex QI intervention works or not, to understanding the causal relationships between contexts and the outcomes of the intervention [20]. A realist review is one approach to uncover some of the contexts and mechanisms that influence the sustainability of Lean. This approach will help address *for whom, under what circumstances, how and why are Lean efforts sustainable or not sustainable in pediatric healthcare?*

### Review question

The purpose of the review was to develop and refine an initial program theory on Lean sustainability in healthcare and to address the research question: *For whom, under what circumstances, how and why are Lean efforts sustainable or not sustainable in pediatric healthcare?* This realist review sought to (a) identify core mechanisms that generate or contribute to the sustainability or non-sustainability of Lean efforts across pediatric healthcare settings, (b) to identify contextual factors triggering core mechanisms, and (c) to contribute to the theoretical development of the sustainability of Lean efforts in pediatric healthcare.

## Methods

### Rationale for realist approach

The review followed established realist guidance [21–24]. Realist synthesis are useful to make program theories explicit by developing testable hypotheses on the mechanisms by which complex interventions are successful or not, and how certain contexts can trigger different mechanisms that in turn generate different outcomes [25, 26]. Interventions such as Lean can have many potential change processes and outcomes that are non-linear and multifaceted in nature and dependent on social context [27]. A realist approach offers methodological strengths to unpack the “black box” of interventions in comparison to traditional synthesis approaches [28]. From a realist standpoint, to understand the effectiveness of an intervention one needs to develop an understanding of the mechanisms (M) and the contexts that affect whether or not they operate (C) in order to generate an outcome (O) (C + M = O) [21]. The terminology used in the review is outlined in Additional file 1.

### Initial program theory development and CMO mapping

A program theory can be used to frame and evaluate how, for whom, why, and under what contexts complex interventions work or not [29]. Prior to this review, we developed our initial program theory on Lean sustainability in healthcare using a multifaceted approach: (1) iterative brainstorming sessions within the review team; (2) realist methodological expertise (see acknowledgements), a scoping search of literature on Lean, QI, and sustainability; (3) use of a Lean operational definition [18]; (4) use of substantive theory (Normalization Process Theory (NPT)) [30, 31] and a sustainability model (National Health Service (NHS) Institute for Innovation and Improvement Sustainability Model (SM)) [32, 33]; and (5) use of the NHS SM definition for sustainability [32], and a comprehensive definition of sustainability [34].

The NHS SM provided process, staff, and organization contextual factors that potentially explain and increase the likelihood of sustainability and continuous improvement [32], while NPT offered insights into the potential mechanisms that promote or inhibit the embedding of complex interventions into routine everyday practice and the likelihood of sustainability [30, 31]. These underpin each of the initial CMO hypotheses from our initial program theory.

Using the context (C) + mechanism (M) = outcome (O) heuristic, our initial program theorizing comprised of mapping the terrain of contexts, mechanisms, and outcomes. Subsequent to that, seven initial CMO hypotheses were formulated; these hypotheses reflect our initial program theory. It became evident that unpacking the causal pathways in implementation are a necessary precursor to theorizing and testing sustainability CMO’s. We hypothesized that outcomes at implementation (e.g., shared understanding, improved team work), the resources provided during implementation (e.g., Lean training), and the scale of implementation (micro, meso or macro), shapes the contexts (e.g., value congruency, high performing teams), mechanisms (e.g., sense-making, staff engagement, empowerment, accountability), and outcomes for the sustainability of Lean efforts. This concept known as the “ripple-effect” is premised on the idea that Lean is a series of “events in the history of a system, leading to the evolution of new structures of interaction and new shared meanings” p. 267 [35]. Our initial program theory depicts that Lean becomes a complex intervention when implemented to a complex adaptive system (healthcare) across multiple levels of a system (micro, meso, and macro) to multiple stakeholders (organizational leaders, clinical leaders, and front-line staff). Our seven initial CMO hypotheses were categorized according to these elements.

### Search methods

Consistent with a realist approach, our search strategy was multi-pronged, iterative, and purposeful in nature. We developed search strategies for the following databases which were searched from date of inception until June 2016: Medline (OVID), EMBASE (OVID), CINAHL (Ebsco), and Dissertation Abstracts (ProQuest). The search strategy consisted largely of keywords since the databases searched did not contain controlled vocabulary for Lean management concepts. Methodological filters were not used, since the goal of a realist review is to identify both qualitative and quantitative reports. We also searched for the term “pediatric” and synonyms in an EndNote database of 5000 references compiled from searches for a systematic review on lean management in healthcare [36].

We conducted reference list searches for each included source. Our gray literature search was purposeful and multi-pronged, undertaken on the following organizational web sites: Institute for Healthcare Improvement (http://www.ihi.org) and the Agency for Health Care Research and Quality (AHRQ) (http://www.ahrq.gov); and Google. All web sites were searched for the terms Lean, healthcare and healthcare synonyms; we scanned the first three pages of Google results. We also contacted three organizations known to implement Lean in healthcare settings: Saskatoon Children’s Hospital, Cincinnati Children’s hospital, and Virginia Mason hospital. Our search strategy is provided in Additional File 2.

### Screening methods and inclusion criteria

Following a two-stage process, two reviewers (RF and SW) independently screened the titles and abstracts of all records (stage 1), and then independently screened the full text (stage 2) of any document that made it through stage 1. For inclusion, documents had to discuss Lean implementation (exclusively or blended, that is Lean and another QI approach) as defined by our operational definition [18], with a pediatric focus (exclusively or blended, that is pediatric and non-pediatric foci in the same study), and sustainability outcomes as defined by NHS SM [33] and Moore et al. [34]. For sustainability outcomes, documents had to provide: (a) measures of sustainability and/or (b) a critique or review of ideas related to how Lean is or is not sustained in pediatric healthcare, and/or (c) stakeholders opinions or accounts of how Lean is or is not sustained in pediatric healthcare. Documents were not excluded based on methodological quality. Due to feasibility reasons, we only included documents in the English language. For stage 1 screening, we applied the inclusion criteria to the titles and abstracts of our search results; all yes and unsure documents moved forward to stage 2 screening, which consisted of full-text screening based upon the inclusion criteria.

### CMO contribution and methodological quality

Adopted from Wozney et al. [37], we assessed each document in terms of the richness and relevance of content to context, mechanism, and outcomes. Each document was rated as low/no contribution (no or little information), medium contribution (some information), and high contribution (well-described information). We also assessed relevance by objective (empirical) versus subjective (anecdotal) evidence. Empirical evidence was determined as research based data (e.g., qualitative or quantitative findings), primarily found in the results section of included documents. A document was classified as providing anecdotal evidence when no empirical evidence supported the author’s interpretations, typically found in the discussion sections of the included documents. We used and adapted the Mixed-Methods Appraisal Tool (MMAT) [38] to assess methodological quality of the included primary studies, resulting in a methodological rating of 0, 25, 50, 75, and 100% (with 100% being the highest quality). We adapted the MMAT for multi-method studies by assessing each segment of a multi-method study separately and then selecting the lowest quality rating. Documents were not excluded based on MMAT score; the purpose was to examine and gain insight into the rigor of existing research in this field. Documents were also not excluded based on anecdotal evidence—our main concern was finding information with strong CMO contribution. This information was logged during data extraction.

### Data extraction

Using a standardized data extraction form on Microsoft excel, we extracted descriptive information from each document (e.g., QI initiative purpose, stakeholder type, setting, theory, and level of change). We applied Colquhoun et al. [39] three conditions for classification of a theoretical basis [39], in order to understand to what extent the sustainability evaluation in each document was guided by theory. We also extracted intervention and contextual factors, mechanisms, outcomes, and any evidence or information related to our initial program theory and CMO hypotheses on the sustainability of Lean efforts. To promote consistency, a coding dictionary was developed and used during data extraction. Two authors (RF and SW) conducted and cross-checked data extraction decisions for each of the included documents. No discrepancies arose during this process. The two authors that conducted data extraction met at after independently completing two extractions and met twice weekly during data extraction.

### Data analysis and synthesis

Data analysis and synthesis were iterative, using a multi-stepped approach to identify and organize information from the included documents. The purpose was to understand what about the contexts where Lean implementation occurred, triggered certain responses (mechanisms) by stakeholders that contributed to the sustainability or otherwise of Lean efforts (outcomes).

Drawing from abductive and retroductive analysis [40, 41], RF examined each document for evidence that supported, refuted, or refined our initial CMO hypotheses. This form of synthesis required the researcher to move between theory and data, analyzing data that were not in the initial program theory (abduction) and moving between theory and observable data (retroduction). This analytical approach enabled the formation of new ideas beyond the initial theoretical basis of our initial program theory and CMO hypotheses and required the researcher to bring assumptions on what factors contribute to Lean sustainability and a priori knowledge on Lean implementation in one health system to question the conditions for a theoretical basis. Retroduction involved inductive and deductive logic where the research team theorized what causal powers may be at play to produce observed patterns in the data. This involved using the teams’ insights and experiences on Lean in healthcare and implementation science. Abductive reasoning involved theorizing the best possible explanations of observed outcomes, thinking about the potential mechanisms and contexts that produced certain outcomes, where data was missing. Data gathered from the included documents that were not explained by our initial CMO hypotheses were used to refine our initial program theory. This process was tracked through reflective notes; integration of NPT and NHS were applicable and through regular team discussion.

## Results

**Fig. 1:**
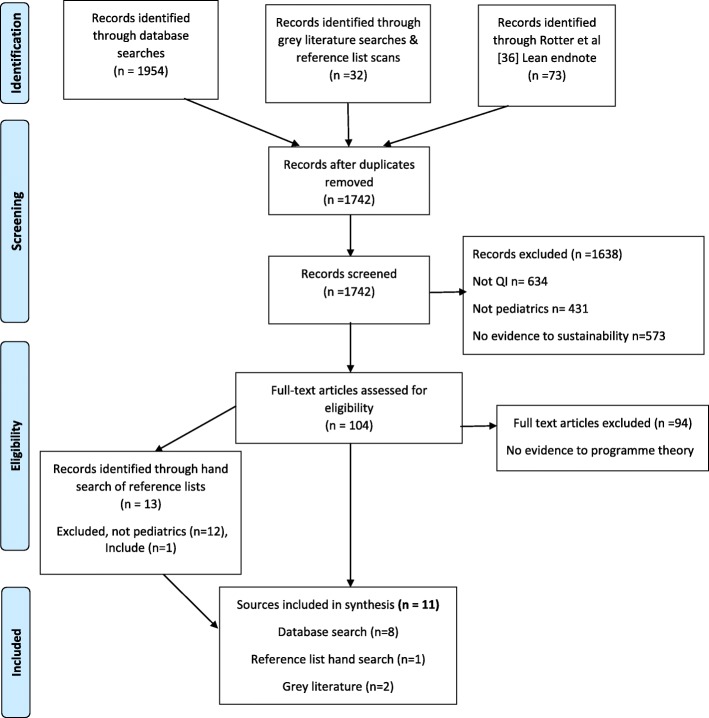
Adapted 2009 PRISMA flow diagram. Search and screening results from the review. From Moher D, Liberati A, Tetzlaff J, and Altman DG, the PRISMA group (2009). Preferred reporting items for systematic reviews and meta-analyses: the PRISMA statement. PLoS Med 6(7): e1000097. doi:10.1371/journal.pmed1000097 [68]

We identified 2059 references from all search methods; 317 were duplicates. We screened titles/abstracts of 1742 documents, reviewed full text of 104, and included 11 documents. We obtained no additional data by contacting Saskatoon Children’s Hospital, Cincinnati Children’s hospital, and Virginia Mason hospital. Eleven documents [42–52] were included in the review and were used to refine the initial CMO hypotheses, eight documents from our database search [42–49], one from our citation search [51], and two documents from our gray literature search [50, 52] (Fig. 1). Results are organized by document characteristics, CMO contribution and methodological quality, sustainability outcomes, evidence in relation to initial CMO mapping and program theory, and finally, the five CMO hypotheses from our initial program theory that were substantively supported by evidence on sense-making and value, staff engagement and empowerment, at the organizational, clinical leadership level and front-line healthcare provider level, and the “ripple-effect” from implantation to sustainability.

### Document characteristics

Of the 11 included documents, six were published primary research studies [42, 43, 45, 47–49], two were published quality improvement case studies [44, 46], and three were unpublished quality improvement case reports [50–52] found from our citation searching [51] and gray literature searching [50, 52] (Table 1). Of the 11 documents, seven used Lean exclusively [44, 45, 48–52]; two used Lean and Six Sigma [42, 43]; one used a combination of Lean, Six Sigma, and the Institute for Healthcare Improvement (IHI) Model for Improvement [46]; and another used Lean with “other” QI classic methods [47]. Improvements were targeted at the meso (e.g., unit or organization level) (*n* = 8) [42–49] and macro (e.g., policy, system) (*n* = 3) [50–52] levels of the healthcare system. No improvement targeted the individual, micro level. Documents focused on a variety of problems, clinical (*n* = 1) [42], process (*n* = 2) [43, 48], clinical and process (n = 1) [46], or process and system problems (*n* = 7) [44, 45, 47, 49–52].

**Table 1:** Document characteristics

Author, year, country	Design	Theoretical framework	QI method and QI purpose	Study purpose	Implementation leaders	Setting and system level
Primary research studies (*n* = 6)						
Tekes, 2015, USA [42]	Pre-post survey	No mention at all	Lean, Six Sigma, clinical	Determine if multi-disciplinary LSS could reduce reliance on head CT in pediatric hydrocephalus population by 50% within 6 months, 24/7.	Multi-disciplinary team, project leader (neuroradiologist), and a physician champion.	Division of pediatric radiology and neuro radiology (meso)
Czulada, 2015, USA [43]	Multi-methods	No mention at all	Lean, Six Sigma, process	Describes the inclusion of a family advisor on an improvement project team to increase communication opportunities.	Multi-disciplinary team, medical director, nurse manager, family advisor.	Pediatric intensive care unit (meso)
Harrison, 2016, USA [45]	Mixed-methods	Explicit statement of theoretical framework and/or constructs applied to the research.	Lean, process and system	Examine how internal organizational context affected the implementation and outcomes of organization-wide Lean initiatives and cycle Lean process redesign projects, were embedded within the “initiatives.”	Senior leadership support, middle management, multi-disciplinary teams, internal or external Lean experts, organizations (added Lean to existing QI practices).	Five organizations, one was a pediatric care continuity (meso).
Northway, 2015, Canada [47]	Multi-methods	No mention at all	Lean and other QI “classic” methods, process and system	Report the long-term sustainability of a transfer protocol.	Multi-disciplinary team, physician and clinical leaders, external Lean experts.	Pediatric intensive care unit (meso).
Mazzacato, 2014, Sweden [48]	Mixed-methods	Explicit statement of theoretical framework and/or constructs applied to the research	Lean, process	Explain how different emergency services adopt and adapt the same hospital-wide Lean-inspired intervention and how this is reflected in hospital process performance data.	Hospital management strategic-hospital-wide Lean-inspired program. Multi-disciplinary improvement teams, internal improvement coaches, physician leaders.	Seven emergency service departments (2 pediatric) (meso)
Mazzacato, 2012, Sweden [49]	Mixed-methods	No mention at all	Lean, process and system	To unpack how and why such a lean application may work.	Multi-disciplinary team, physician lead, internal process improvement coaches, hospital management.	Pediatric emergency unit (meso).
Quality improvement reports (*n* = 5)						
Wong, 2016, Canada [44]	Commentary/descriptive	No mention at all	Lean, process and system	Illustrate how an implicit mental model pervades in the healthcare system based on deeply held but unexamined assumptions that arise from heuristics and biases, that can be examined by objective data and how we can build a new mental model.	Multi-disciplinary team, process improvement team and senior hospital management support.	Pediatric eye clinic (micro).
Luton, 2015, [46]	Commentary/descriptive	No mention at all	Lean, Six Sigma, IHI Model for Improvement, clinical and process	To describe how a program to prevent feeding errors was developed, implemented, and evaluated.	Multi-disciplinary team, QI project manager, executive task force support (leaders).	Newborn center (three discrete NICUs, milk bank, and formula room) (meso)
Carman, AHRQ, 2014, USA [50]	Commentary/descriptive	No mention at all	Lean, process and system	To examine the ways in which each organization has implemented Lean and identify the factors that influenced progress within individual Lean projects and on the ultimate outcomes.	Executive managers, CEO, clinical managers, external Lean consultants, management engineers, and multi-disciplinary front-line teams.	Five case studies of organizations that implemented Lean-blended adult and pediatrics. Case 1, four hospitals, 3 are pediatrics (macro)
Hung, AHRQ, 2016, USA [51]	Multi-methods	Explicit statement of theoretical framework and/or constructs applied to the research.	Lean, process and system	Study the scaling and sustainability of Lean redesigns as an organization wide initiative, with a particular focus on analyzing contextual factors affecting the success of implementation efforts.	Ambulatory care system-wide Lean initiative, executive leadership, external Lean consultants, clinical leaders, physicians and multi-disciplinary front-line staff.	Ambulatory care system with primary care departments (includes pediatrics) across Palo Alta Medical Foundation (macro)
Rotter, 2014, Canada [52]	Multi-methods	Explicit statement of theoretical framework and/or constructs applied to the research.	Lean, process and system	Evaluate the early stages of the implementation of Lean (Saskatchewan’s Lean Management System) in the provincial health system.	Ministry strategy policy makers, executive management support, external Lean consultants, clinical leaders, Kaizen promotion office, multi-disciplinary teams.	Saskatchewan Healthcare System (twelve regions)–focus on four regions for realist evaluation (pediatric data) (macro)

Legend of the information extracted, four levels of change in health system: the individual (micro level), the group or team, the organization (meso level), and the larger system or environment (macro level) in which individual organizations are embedded [70, 71]. Clinical: (a) involving direct observation of the patients’ clinical diagnosis, (b) based on or characterized by observable and diagnosable symptoms clinical treatment [73]. Process: A series of actions or steps (procedures) taken in order to achieve a particular end (outcome) [74]. System: (a) a set of detailed methods, procedures, and routines created to carry out a specific activity, perform a duty, or solve a problem (b) an organized, purposeful structure that consists of interrelated and interdependent elements (components, entities, factors, members, parts, etc.)

These elements continually influence one another (directly or indirectly) to maintain their activity and the existence of the system, in order to achieve the goal of the system [74]. Theoretical framework: no mention at all, reference to broad theoretical basis, reference to specific theoretical basis, explicit statement of theoretical framework and/or constructs applied to the research [34]

All documents used a multi-disciplinary team approach to lead implementation [42–52], six of which also included a physician lead within the multi-disciplinary team [42, 43, 47–49, 51]. Many reported organizational leadership involvement (*n* = 8) [44–46, 48–52] and/or clinical leadership involvement (n = 7) [43, 45–47, 50–52]. One reported patient involvement [43]. There was variation between the use of internal QI support coaches [44, 45, 47] versus external Lean experts or consultants [48–52].

### CMO contribution and methodological quality

The methodological quality (Table 2) of the six primary research studies varied, three scored 75% [42, 48, 49], two scored 25% [45, 47], and one had no score, 0% [43] on the MMAT. Relevance (CMO contribution) also varied across the primary research studies. Two studies with an MMAT of 75% had high contribution [48, 49]; however, in contrast, the third study with an MMAT of 75% had low contribution [42]. One study that scored 25% on the MMAT had a high contribution [45]. The remaining two studies with MMAT scores of 25% and 0% had medium contribution [43, 47]. Of the quality improvement case reports (*n* = 5), four had medium contribution [44, 46, 50, 51] and one had high contribution [52].

**Table 2:** CMO contribution and methodological quality

Published primary research studies (*n* = 6)					
Author, year, country, citation	Design	MMAT score	Objective versus subjective data	CMO contribution level	Theory
Tekes, 2015, USA [42]	Quantitative descriptive (pre-post survey)	75%	Objective data	Low	None
Czulada, 2015, USA [43]	Multi-methods	0%	Objective data	Medium	None
Harrison, 2016, USA [45]	Mixed-methods	25%	Objective data	High	CFIR
Northway, 2015, Canada [47]	Quantitative descriptive	25%	Objective data	Medium	None
Mazzacato, 2014, Sweden [48]	Mixed-methods	75%	Objective data	High	Realist
Mazzacato, 2012, Sweden [49]	Mixed-methods	75%	Objective data	High	None
Published quality improvement case studies (*n* = 2)					
Wong, 2016, Canada [44]	QI project commentary/descriptive	n/a	Subjective data	Medium	None
Luton, 2015, USA [46]	QI project commentary/descriptive	n/a	Subjective data	Medium	None
Unpublished quality improvement case report (*n* = 3)					
Carman, AHRQ, 2014, USA [50]	Case report commentary/descriptive	n/a	Objective data	Medium	None
Hung, AHRQ, 2016, USA [51]	Case report Multi-methods	n/a	Objective data	Medium	CFIR
Rotter, 2014, Canada [52]	Evaluation report Multi-methods	n/a	Objective data	High	Realist

Methodological quality of the included primary studies was assessed using Mixed-Methods Appraisal Tool (MMAT) [33]. Each document was rated as low/no contribution (no or little information), medium contribution 28 (some information), and high contribution (well-described information) for context, mechanism, and outcomes contribution [32]

### Sustainability outcomes

There was variation as to how sustainability was defined and measured. For example, six documents referred to sustainability as a change that had lasted over a certain period of time ranging from 6 months to 4 years [42, 43, 46–49]. Outcome measurements were not widely reported, and the description on sustainability was poor, primarily consisting of descriptive and experiential accounts (e.g., “Long-term sustainability requires staff engagement, charismatic champions and leaders, and a culture that sustains the change despite staff turnover”) [47]. Of the six primary research studies, three reported positive sustainability outcomes [42, 43, 49], and three reported mixed (positive and negative) sustainability outcomes [45, 47, 48].

All the primary research studies reported clinical, process, and performance outcomes as the proxy measure for sustainability [42, 43, 45, 47–49]. For example, one study reported, “process changes were implemented, resulting in an increased mean documented communication rate from 13% pre intervention to 65% post intervention that was sustained for more than 2 years (P<.001)” [43]. One of the studies that reported mixed outcomes stated that, “we lack hard data on these measurable outcomes of their long-term sustainability” [45]. The same study reported some negative outcomes that the implementation of Lean had short-term gains and failed to achieve more widespread and sustained improvements; these data was gathered through qualitative interviews [45]. Outcomes reported from the remaining included documents were based on subjective data from descriptive QI reports [44, 46], or case study reports that had collected primary objective data but presented summary findings [50–52].

### Examining the evidence in relation to initial CMO mapping and program theories

By using a realist approach, we have been the first to uncover some of the contexts and mechanisms that influence the sustainability of Lean efforts in pediatric healthcare. Three substantial issues have emerged and have supported our initial program theory.

First, the degree of success or failure in the sustainment of Lean efforts relies on the ways in which people “make sense” of Lean, align their values, and the values of the organization to the values of Lean. Sense-making (the process through which people assigns meaning to experience), staff engagement, and empowerment were identified as core mechanisms to the sustainability or non-sustainability of Lean efforts. The activation of these mechanisms was facilitated or hindered by Lean resources, such as Lean education [42, 46, 47, 50], Lean training [43, 45, 49, 50, 52], external Lean consultants [45, 48–52], internal QI support coaches [44, 45, 47], and knowledge translation strategies [42, 43, 47]. The degree to which these mechanisms were activated or not was influenced by certain conditions or contextual factors, such as external pressures to use Lean [43, 45, 46], a culture shift prior to implementation (organizational readiness) [44], an existing QI structure [43, 44] staff turnover [45, 47, 48], the silo nature of healthcare [49], the complexity of care processes [48], the fit between Lean and local context [47, 48], and other competing needs or demands [47]. It is important to note that none of the contextual factors identified were unique to pediatric contexts. The relationship between these contexts and mechanisms led to multiple heterogonous outcomes on the sustainability of Lean efforts.

Second, outcomes from Lean implementations shifted to become the contexts for sustainability. That is, in some cases, there was a “ripple-effect” where outcomes from implementation served as facilitating or hindering contexts that triggered mechanisms for the sustainment or otherwise of Lean efforts. For example, “sense-making” and value congruency are outcomes at implementation that serve as contexts in sustainability which then trigger staff engagement and empowerment to lead and sustain Lean efforts. Hence, the efforts taken and approaches used at implementation are critical to the success of sustaining Lean efforts.

Implementation approaches and processes contributed to the sustainability or non-sustainability of Lean efforts across our included documents. The use of multi-disciplinary-led teams [42–52], patient involvement [43], physician leads [42, 43, 47–49, 51], organizational leadership involvement [44–46, 48–52], and/or clinical leadership involvement [43, 45–47, 50–52] contributed to the sustainability of Lean efforts. For example, large-scale transformation was reported to have greater likelihood of sustainability than small-scale incremental QI improvements [48], with top-down leadership commitment [44]. However, it was noted in another document that a top-down implementation approach was less well received and sustained [51]. These contradictory findings demonstrate that top-down approach was equivocal in terms of sustainability. External Lean consultants were also reported as a facilitator to sustainability [48].

Finally, Lean is complex in the context of healthcare; its implementation and sustainability are complex as it occurs across multiple levels of organizations [53] within complex adaptive systems [54] with multiple stakeholders. Contexts, mechanisms, and outcomes at one layer of a health system (e.g., organizational leadership) had an impact on the contexts, mechanisms, and outcomes at another level (e.g., clinical leadership), demonstrating the need for a theoretical complexity lens to the implementation and sustainability of Lean in healthcare.

### Substantially supported CMO hypotheses

#### Value and vision congruency, sense-making as motivations to sustain lean efforts

##### CMO hypothesis 1

*If the values of organizational leaders are congruent with Lean philosophy, and leaders receive Lean leadership training (C), then organizational leaders are more likely to make-sense of, appreciate, and feel motivated to implement Lean (M), in turn, they become Lean messengers, promoting Lean philosophy to clinical leaders of the organization (O).*

Six documents [42, 44, 45, 48, 49, 51] substantiated our initial CMO hypotheses that value congruency and coherence between all levels of the organization, and Lean philosophy and activities are critical to the sustainability of Lean efforts. Contexts where, Lean “fits”, makes sense and aligns with the values of the organization in its entirety, and the people that make up that organization are critical to sustainability. For example, three documents reported that Lean value congruency should begin at the organizational level, “where clear goals/vision aligned with institutional and departmental priorities and mission” [42] and “where there is top-down commitment, where CEOs and senior executives need to understand and embrace Lean thinking by integrating it into their philosophy and operating strategy” [44]. A third document reported that, “the degree to which leaders aligned the Lean initiative with their organizational vision had important consequences for the overall initiative and for projects embedded within it” [44].

##### CMO hypothesis 4

*If there is congruency between Lean philosophy and the personal-level reasoning of the clinical leaders and front-line healthcare providers, and clinical leaders and front-line healthcare providers receive Lean leadership training (C), then Lean is more likely to make sense and fit within the context (M), in turn, motivating clinical leaders to become Lean messengers, promoting Lean philosophy to front-line staff (O).*

Three included documents supported our initial CMO hypotheses that the processes of value congruency and sense-making are contexts that trigger either a positive or negative behavioral response by stakeholders, resulting in the outcome of sustained Lean efforts [45, 48, 49]. One document reported, “clinical staff who believed that the overriding purpose of Lean was cost cutting- rather than improving patient experience”; these participants saw Lean as an added burden to their work [45]. Another discussed issues of differing values and the experience of conflicting loyalties for process leaders between hospital management and their department and how this lack of strategic alignment hindered institutionalization of Lean changes [48]. In another document, Lean efforts made-sense and created standardized work and clear roles for some staff, but for others, this approach made them feel their work was more narrowly regulated [49]. Contexts where there was a team approach to Lean activities facilitated different professions (e.g., nurses and physicians) to make sense of each other’s work and how their work related to that of others and patient needs [49].

In relation to important contextual factors required to sustain Lean efforts, one document reported that initial specific education to establish a common language and way of thinking about QI was critical to the success of integrating these processes into the culture [46]. Also, in relation to context, one document had some senior leaders report that process improvement was already a part of their organizations culture, thus introducing Lean was not foreign to staff; however, others argued that some staff did not understand how Lean was integrated into the larger QI strategy in the organization [51] potentially causing a lack of congruency and the need for continued sense-making activities (e.g., education, training, messaging).

#### Front-line staff engagement and empowerment as mechanisms to sustain Lean efforts

##### CMO hypothesis 6

*If contexts exist where staff are engaged, have received Lean training and the opportunity to lead Lean efforts (C), then staff are more likely to become empowered to use Lean (M), and can then see beneficial outcomes from Lean, have improved satisfaction leading to increased sustained use of Lean efforts(O).*

Seven documents reported that engaging healthcare professionals in designing, overseeing, and managing their own processes and opening new lines of communication through the hospital hierarchy was a contributor to the context of sustainability of Lean efforts [44–48, 50, 52]. Engagement was triggered through active input from front-line staff on things that were important to them, aligning their values with Lean [45]. For example, one document reported that, “Lean activities enabled staff to provide input into redesigning processes that were important to them. Employees grew more satisfied because of improvements in patient experiences, employee collaboration, efficiency, and opportunities to spend more time with patients” [45]. Staff engagement was sustained by soliciting their ideas at the end of their shifts [44]. Integrated multi-disciplinary staff engagement broke down silos [40], build trust, and improved communication channels [46, 49–51]. However, a multi-disciplinary team approach did not always work well, with some professions feeling a sense of unwillingness to work together from another [49]. As a counter theory, lack of congruency between values was reported as barrier to engagement, where there was poor alignment between the problems identified and the changes introduced [48].

##### CMO hypothesis 7

*If there are contexts where there are visible benefits from Lean implementation, and a collaborative multi-disciplinary team approach to Lean implementation, with audit and feedback of changes (C), this triggers staff motivation and empowerment to sustain Lean efforts (M), then Lean efforts become integrated and sustained in practice (O).*

Engagement was reported as a “trigger” for staff empowerment [49]; staff who was more engaged felt more empowered. Staff empowerment was reported in four documents [44, 49, 50, 52]. In one document, it was hypothesized that Lean empowers staff in contexts where there is multi-disciplinary participation in the application of Lean tools, and that staff empowerment itself then operates as a mechanism for improved patient safety [52]. Empowerment was triggered through reflective time and the authority to identify and eliminate waste [44, 50]. Another found that the team approach empowered front-line staff to manage and share ideas for improvement [49]. However, others in the same study reported that changes were occurring too fast with a frustrating amount of numerous modifications to care processes [49], this could be a sense of “innovation fatigue.”

### Ripple-effect

**Fig. 2:**
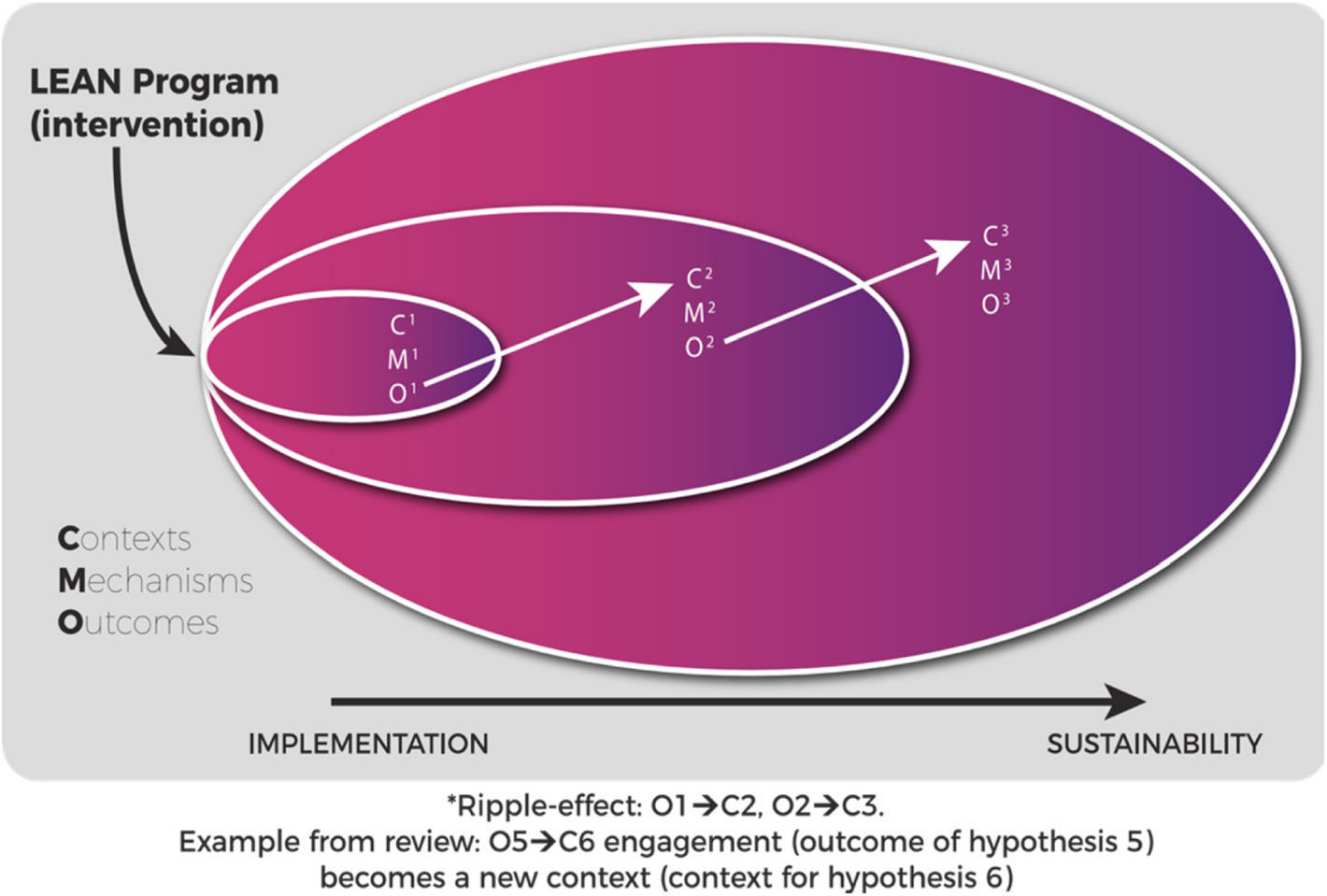
Ripple-effect graphic. CMO ripple effect, where the outcome from one CMO serves as the context to the subsequent CMO, for example, O1 becomes C2

The concept of the “ripple-effect” enabled a better understanding of the causal relationship between Lean implementation and sustainability, and how processes from implementation to sustainability occur. Our review findings demonstrated instances where outcomes of Lean implementation served as facilitating contexts for subsequent stages of sustainability (C^1^M^1^O^1^– > C^2^M^2^O^2^) [55] as illustrated in Fig. 2. This concept of a “ripple-effect” was substantiated in CMO hypothesis 5 and 6, where staff engagement was an outcome at implementation, in turn a context for sustainability, which triggered mechanisms of staff empowerment and outcomes of sustained Lean efforts.

## Discussion

Lean has been implemented in several pediatric settings and healthcare systems in the absence of an understanding as to why (or why not) it works, how it works or not, for whom and in what contexts. This lack of evidence can negatively impact the likelihood of sustaining Lean efforts. Sustainability was not an exclusive focus in the documents included in this review. Rather, it was an evaluation aspect of successful Lean implementation. Typically, sustainability was referred to as a “point of time” or through process, performance and clinical outcome measures to Lean sustainability. Similarly, a scoping review of 43 studies on Lean management in adult-only healthcare settings [36] identified a lack of reporting on the follow up and sustainability of Lean implementation. Some of the evidence sourced in our review was experiential or anecdotal. This echoes Greenhalgh and colleagues [56] who suggested that there is a dearth of studies which focus on the sustainability of complex service innovations. Also, supported by our review findings, there is heterogeneity in the literature on how sustainability is conceptualized [16, 17], and the timeframes appointed to measure sustainability outcomes [16].

In our review, only four documents used formal theory [45, 48, 51, 52], all of which were implementation theories [45, 51] or realist evaluations [48, 52]. None of the documents in this review were underpinned by a sustainability theoretical framework, model, or measurement tool. There is a lack of conceptual models and frameworks on sustainability [16, 17], a recognized priority but challenging area for future research, where it is unknown if the predictors of implementation and sustainability are the same or different from each other [16].

### Sense-making and value congruency

As demonstrated through this review, sense-making about Lean may occur at implementation but is also crucial to sustainability, if the philosophy, principles, and activities of Lean do not make sense to those tasked with implementing or using Lean, then it is unlikely that Lean efforts will be adopted and subsequently sustained. This finding supports and substantiates CMO hypotheses 1 and 4 from our initial program theory (Table 3.). The more people value the change being implemented as important or worthwhile, the more likely that they will engage in the implementation efforts [53]. Some empirical evidence has shown that when staff and managers did not understand Lean, this had a negative meaning throughout the organization [54]. Sense-making is associated with productive self-organization [57], a process whereby natural order forms irrespective of the interventions intentions [58]. Creating and maintaining an institutional culture underpinned by shared vision and values are central to Lean success [59]. Supportive culture with leadership engagement and team involvement was an identified facilitator to maintaining Lean efforts [13], demonstrating that engagement must occur across different layers of the organization.

**Table 3:** Initial program theory development work: CMO mapping and hypotheses

System level: organizational leadership level (macro or meso)			
CMO hypothesis 1: *If the values of organizational leaders are congruent with Lean philosophy, and leaders receive Lean leadership training (C), then organizational leaders are more likely to make-sense of, appreciate, and feel motivated to implement Lean (M), in turn, they become Lean messengers, promoting Lean philosophy to clinical leaders of the organization (O).*			
Context (C1)	Mechanism (M1)	Outcome (O1)	Link to formal theory
The degree of congruency between Lean philosophy and the values of the organizational leaders and the extent of other contextual forces (e.g., political and economic environments). The degree and nature of Lean leadership training for organizational leaders.	The degree of sense-making about how Lean is relevant to an organization. Realization of the fit between the Lean philosophy and the organizations vision and/or mandate. The degree of appreciation of the Lean philosophy from organizational leaders.	The extent of Lean capacity building at top level of an organization. The extent to which organizational leaders are motivated to be “Lean leaders” and “Lean messengers.” *Messaging efforts* The extent to which organizational leaders use their influence to promote “message” Lean to clinical leadership. *Ripple-effect 01➔ C2 *Messaging efforts* (outcome of hypothesis 1) becomes a new context (context for hypothesis 2)	NHS SM organization factor 9: fit with the organization’s strategic aims and culture. NPT coherence internalization: understanding the value, benefits, and importance around a set of practices. NPT coherence individual specification: participants need to do things that will help them understand their specific tasks and responsibilities around a set of practices.
CMO hypothesis 2: *If there are strong “messaging” efforts from organization leaders in promoting Lean, in a way that resonates with clinical leaders and front-line staff (C), then people are more likely to see value in Lean, gain a shared cohesive understanding of Lean benefits throughout the organization (M), thus creating increased buy-in and engagement to Lean efforts(O).*			
Context (C2)	Mechanism (M2)	Outcome (O2)	Link to formal theory
*Ripple-effect 01➔ C2 The degree of messaging about the value and purpose of Lean by organizational leaders to the wider organization. The degree of congruency between Lean philosophy and personal-level reasoning of the clinical leaders and front-line healthcare providers. The degree of credible and respected senior leaders are seen as promoting and investing their own time in Lean efforts.	The nature of how organizational leaders promote “message” Lean (i.e., “you have to do it,” or “that is a new mandate”) (resource) will trigger a degree of receptivity and value (positively or negatively) by the clinical leaders and front-line staff.	The degree of shared understanding about Lean benefits. The degree of buy-in, uptake, and engagement for doing Lean activities by organizational leaders, clinical leaders, and front-line staff.	NPT coherence communal specification: sense-making relies on people working together to build a shared understanding of the aims, objectives, and expected benefits of a set of practices. NPT cognitive participation initiation: when a set of practices are new or modified, a core problem is whether or not key participants are working to drive them forward. NHS SM staff factor 7: senior leadership engagement.
CMO hypothesis 3: *If front-line staff believe that Lean is more than the “latest quality improvement trend” in a context with good staff morale and continued resources for Lean efforts (C), then front-line staff will have greater trust and belief in the long-term benefits of Lean (M), resulting in improved Lean implementation and positive influences for a continuous quality improvement culture (O).*			
Context 3 (C3)	Mechanism 3 (M3)	Outcome 3 (O3)	Link to formal theory
The degree of staff turnover, staff morale, type of unit culture, and level of innovation fatigue. The extent of time, continual resources (e.g., staff, facilities, equipment, policies, and procedures), and staff capacity (training, audit and feedback, communication channels, senior leadership support) provided for sustainability.	The extent to which stakeholders in the organization believe that Lean is there for the long-term and not just the “latest quality improvement trend” (response). The degree of trust built by front-line staff in the Lean approach, the changes taking place, and the support from leadership.	Inefficiencies or efficiencies in Lean implementation. Increased workload (i.e., stress, burnout) or supported workload (i.e., reduced stress, burnout). Frustration or satisfaction with Lean efforts. Facilitated or hindered culture for continuous quality improvement.	NHS SM organization factor 10: infrastructure for sustainability.
System level: clinical leadership level (meso)			
CMO hypothesis 4: *If there is congruency between Lean philosophy and the personal-level reasoning of the clinical leaders and front-line healthcare providers, and clinical leaders and front-line healthcare providers receive Lean leadership training (C), then Lean is more likely to make sense and fit within the context (M), in turn, motivating clinical leaders to become Lean messengers, promoting Lean philosophy to front-line staff (O).*			
Context 4 (C4)	Mechanism 4 (M4)	Outcome 4 (O4)	Link to formal theories
The degree of congruency between Lean philosophy and personal-level reasoning of the clinical leaders and front-line healthcare providers. The degree of Lean training received by clinical leaders and front-line healthcare providers.	The degree of sense-making process to understand how Lean is relevant for practice and patient care. Realization of the extent in which Lean philosophy fits to their particular health care context or mandate. The degree of appreciation of Lean philosophy from clinical leaders and front-line healthcare providers.	The extent to which clinical leaders are motivated to be “Lean leaders” and “Lean messengers.” The extent to which clinical leaders use their influence to promote “message” Lean to front-line staff. *CMO1 and CMO4 are about messaging efforts; however, the nature of the messaging may be different at different levels of systems	NPT coherence internalization: understanding value, benefits, and importance around a set of practices. NPT coherence individual specification: participants need to do things that will help them understand their specific tasks and responsibilities around a set of practices.
CMO hypothesis 5: *In contexts where there are positive relationships between the clinical leader and front-line staff, and clinical leaders play an active role in Lean implementation (C), then front-line staff are more likely to believe in their leader’s commitment to Lean, engage in Lean activities themselves (M), leading to buy in and continued support of Lean efforts (O).*			
Context (C5)	Mechanism (M5)	Outcome (O5)	Link to formal theories
Competing demands on clinical leader and their workload, affecting time commitment Lean. Positive or negative relationships (e.g., trust, communication) between clinical leader andfront-line staff. Leadership approach used by clinical leaders’ (hierarchical versus distributive). The degree that clinical leaders play active role in promoting, participating, and investing own time in Lean assessment and improvement activities.	The extent to which front-line staff believe in managers’ commitment to Lean. The degree of front-line staff feeling engaged.	The extent of continued buy-in and engagement by front-line staff. The degree of continued input and support of Lean efforts and use of Lean activities. *Ripple-effect O5➔C6 engagement(outcome of hypothesis 5) becomes a new context (context for hypothesis 6).	NHS SM staff factor 8: clinical leadership engagement.
System level: front-line healthcare provider level (micro or meso)			
CMO hypothesis 6: *If contexts exist where staff are engaged, have received Lean training and the opportunity to lead Lean efforts (C), then staff are more likely to become empowered to use Lean (M), and can then see beneficial outcomes from Lean, have improved satisfaction leading to increased sustained use of Lean efforts(O).*			
Context 6 (C6)	Mechanism 6 (M6)	Outcome 6 (O6)	Link to formal theories
*Ripple-effect 05➔ C6 Engaged staff. Core values of front-line healthcare providers that align or impede their motivation; pre-existing levels of feeling empowered; pre-existing levels of work satisfaction; pre-existing attitude and buy-in of clinical leader; and pre-existing relationships between clinical leader and front-line healthcare providers. Level of morale in the department. Silo or collaborative nature of the system, degree of relationships and collaboration between various stakeholder professions. The degree of Lean training that front-line staff receive and are given the opportunity to drive or lead Lean efforts at the unit level.	The degree to which front-line staffs’ ideas are considered, and opportunities that they are given to test these ideas and their belief that Lean is a better way of doing things and should be sustained. The degree of engagement triggers empowerment of front-line staff in Lean efforts (co-production of Lean customization to local contexts with front-line healthcare providers).	The extent to which benefits to patients, staff, and the organization due to Lean implementation are visible; leading to increased satisfaction and increased sustainability of Lean activities over time. Level of satisfaction, motivation, and commitment by staff. The degree of sustained Lean efforts.	NPT Cognitive participation enrollment: participants may need to reorganize themselves and others in order to collectively contribute to work involved in new practices. This is complex work that may involve rethinking individual and group relationships between people and things. From MHS SM staff factor 5: staff involvement and training to sustain the process. NHS SM staff factor 6: staff attitudes towards sustaining the change. NPT cognitive participation: legitimation: ensuring that other participants believe it is right for them to be involved and that they can make a valid contribution to it.
CMO hypothesis 7: *If there are contexts where there are visible benefits from Lean implementation, and a collaborative multi-disciplinary team approach to Lean implementation, with audit and feedback of changes (C), this triggers staff motivation and empowerment to sustain Lean efforts (M), then Lean efforts become integrated and sustained in practice (O).*			
Context 7 (C7)	Mechanism 7 (M7)	Outcome 7 (O7)	Link to formal theories
The extent to which the benefits to patients, staff, and the organization due to Lean implementation are visible. The degree of collaborative team building and multi-disciplinary team approach to Lean activities.	The degree of “healthy” audit and feedback loops, communication of outcomes. The degree staff feel heard, believe in Lean outcomes, and feel engaged and empowered to sustain Lean efforts.	The extent of Lean integration to everyday practice. The degree of sustained Lean efforts.	NHS SM process factor 2: credibility of the benefits. NHS SM process factor 1: benefits beyond helping patient.

Another challenge with implementing and subsequently sustaining Lean efforts as intended was the dichotomy between the vision and values of Lean with those of the organization and/or key stakeholders within an organization. Without alignment of vision and values from senior leadership to the front-line teams, Lean may be reinterpreted and reshaped to ensure that it fits with the visions and values of the multiple stakeholders, which may also vary, making Lean efforts a highly contested process [10]. Under such conditions with potential conflict and disagreement, Lean efforts are unlikely to be maintained as originally intended. For example, Kim et al. [60] reported the misunderstanding of Lean as a cost-cutting measure, created fear in staff and disengagement in Lean. Another study reported on the overuse of “Japanese” terminology for Lean principles and activities by external Lean consultants in healthcare that do not resonate with health professionals or a patient centered approach. The authors from this study suggest the need to appeal to the personal values and reasoning of the potential adopters [61]. Another published paper on the “promise of Lean in health care” reported that Lean needs to be seen as a mindset that governs how one looks at the business or process. Human skills such as communication, problem solving, teamwork, and strong leadership are vital for Lean implementation success. It is resolute that organizational culture and poor change management are predominant reasons for Lean failures [62].

Active multi-disciplinary staff involvement in leading Lean efforts was recognized as critical to sustainability. This finding is supported by Lean literature in other healthcare contexts [6, 7, 13, 54]. Leadership support was also found in our review to be critical to sustaining Lean efforts, a finding that is shared across other published Lean healthcare literature [4, 11, 59, 62, 63]. Despite the recognition of the importance of these concepts to the embedding, normalization or sustainability of Lean efforts, there is a paucity of rigorous literature that explores or tests these concepts in Lean in healthcare.

### The “ripple-effect”

We hypothesized that outcomes at implementation (e.g., shared understanding, collaborative improved team work collaboration), contextual factors at implementation (e.g., external Lean consultants versus internal QI coaches), and the scale of implementation (micro, meso or macro), shapes the contexts (e.g., value congruency, high performing teams), mechanisms (e.g., sense-making, staff engagement, empowerment, accountability), and outcomes for the sustainability of Lean efforts. This supports the argument by Pluye and colleagues [64] that program implementation and sustainability are not distinct processes but are connected to each other. However, other existing evidence has shown that the conditions that facilitate implementation may diminish overtime [65, 66]; hence, the conditions for sustainability are also susceptible to losing presence and influence, leading to discontinuation.

### Strengths and limitations

A limitation of this review is that we only sourced 11 relevant documents. This paucity of literature demonstrates a knowledge gap and weak evidence base on Lean sustainability in pediatric healthcare. The quality of the literature that served the basis of this review must also be acknowledged as a limitation, making unpacking CMO hypotheses challenging. There is a clear need for more rigorous evaluative studies on Lean sustainability in healthcare. We experienced positive reports about Lean, however, not always based on rigorous research design and method. Theorizing during this process came from expertise on Lean in healthcare, healthcare improvement experts and implementation scientists. Data from this review do support some CMO hypotheses from our initial program theory. A strength is that the results of this realist review are being tested in a subsequent realist evaluation on the sustainability of Lean efforts across four pediatric acute care units at one hospital in Saskatoon, Saskatchewan, Canada. Our realist evaluation takes place in a health system-wide transformation of Lean known as “the Largest Lean transformation in the world” [67]. This is an important next step to test our initial program theory and substantiated CMO hypotheses. A limitation of much of the research on Lean in healthcare and thus, this review, is the lack of reporting and measurement on sustainability.

## Conclusions

This is the first realist review on the sustainability of Lean, a widely implemented complex QI intervention across health systems worldwide. This review demonstrates instances of a causal pathway between implementation and sustainability and a “ripple-effect” from implementation to sustainability. Our findings also demonstrate that sense-making and value congruency during implementation are important contextual factors that trigger the likelihood of sustained Lean efforts. Engagement served as an outcome at implementation and shaped contexts for sustainability as demonstrated through the “ripple-effect.” Empowerment was an important mechanism that triggered the likelihood of sustained Lean efforts. This review also shows that there are many evidence gaps in relation to the sustainability of Lean efforts, and that there is a need for rigorous research to evaluate the underlying factors influencing the success and sustainability of Lean across different healthcare settings.

It remains unknown how a complex QI intervention or program like Lean goes from implementation to normalized behavior, where sustainability efforts are no longer required or have ceased. It is also unknown what about Lean is most important to sustain, what about Lean efforts are sustained in reality (e.g., Lean activity, practice change, culture change), and how to measure the success of Lean efforts in terms of sustainability. There is a need for the development and pilot testing of program theories and tools to evaluate the sustainability of complex interventions in healthcare. Sustainability research on healthcare improvement interventions is critical to enable better implementation, measurement, and reporting. There is a need for further exploration into the mechanisms found in our review and what they mean for sustaining complex QI interventions. We are testing and further refining these mechanisms in pediatric healthcare contexts through a realist evaluation.

## Additional files

Additional File 1: Explanation of used terminology for review. Definitions and descriptions for each key concept used in the review [68–72]. (DOCX 30 kb)

Additional File 2: CINAHL search strategy. Search applied for the review. (DOCX 32 kb)

## Abbreviations

CMO: Context, mechanism, outcome
MMAT: Mixed-Methods Appraisal Tool
NHS SM: National Health Service Institute for Innovation and Improvement Sustainability Model
NPT: Normalization Process Theory (NPT)
QI: Quality improvement
