# Supplementary material for: The sustainability of Lean in pediatric healthcare: a realist review
Source: Syst Rev. 2018 Sep 11;7:137. doi: 10.1186/s13643-018-0800-z (PMC6134523; doi:10.1186/s13643-018-0800-z)
Supplement: Supplementary file 1 — Explanation of used terminology for review. Definitions and descriptions for each key concept used in the review [68–72]. (DOCX 30 kb) [file 13643_2018_800_MOESM1_ESM.docx]

**Additional File 1 Explanation of used terminology for review**

| **Terminology** | **Explanation** |
| --- | --- |
| Realist Methodology | A theory driven, interpretative approach to uncovering underlying middle-range theories (or logics) driving interventions and their multiple components, as well as illuminating the contextual factors that influence mechanisms of change to produce outcomes [55, pg 3]. |
| Programme theory & refined programme theory | Refers to a variety of ways of developing a causal modal linking programme inputs and activities to a chain of intended or observed outcomes, and then using this model to guide the evaluation [29, pg 30].  A ‘refined theory’ is the product of a realist review. In the process of conducting a review, some aspects of the initial rough theory may have been proved wrong.  Others may have been supported with strong evidence.  Many (perhaps most) will have been refined to some extent [23, pg 11]. |
| Middle-range theory | An implicit or explicit explanatory theory that can be used to explain specific elements of programs or how program logic manifests in implementation. “Middle-range” means that it can be tested with the observable data and is not abstract to the point of addressing larger social or cultural forces (i.e., grand theories). MRT is sought at the outset and examined iteratively throughout the review [55, pg 3]. |
| Context-mechanism-outcome (CMO) configurations | CMO configuring is a heuristic used to generate causative explanations about outcomes in the observed data. A CMO configuration may be about the whole program or only to certain aspects. One CMO may be embedded in another or configured in a series (ripple effect in which the outcome of one CMO becomes the context for the next in the chain of implementation steps). Configuring CMOs is a basis for generating and/ or refining the theory that becomes the final product of the review [55, pg 3]. |
| Context | Context can be defined as all factors that are not part of the program or intervention itself, the “backdrop” to implementation, yet does interact, influence, modify, facilitate or hinder the intervention and its effectiveness (in our case the sustainability of Lean efforts) [69]. |
| Mechanisms | Mechanisms are the combination of resources (intended and unintended) offered by a social program under study (Lean) and the response to those resources (cognitive, emotional, motivational reasoning etc.) by stakeholders [21]. Mechanisms will only activate in the right conditions (contexts). |
| Outcomes | Outcomes are a result of a programs firing multiple mechanisms which have different effects on different subjects in different situations, and so produce multiple outcomes. Realist evaluators examine outcome patterns in a theory testing role. Outcomes are analyzed to discover if conjectured mechanism/context theories are confirmed [21, pg 217]. |
| Sustainability definitions | The continuation or the integration of new practice within an organization whereby it has become a routine part of care delivery and continues to deliver desired outcomes, whereby the ways of thinking and attitudes behind processes and outcomes have changed and the new practice has become the new way of working” [32, pg 2].  A comprehensive definition of sustainability which includes five constructs: (1) after a defined period of time, (2) the programme, clinical intervention, and/or implementation strategies continue to be delivered and/or (3) individual behavior change (i.e., clinician, patient) is maintained; (4) the program and individual behavior change may evolve or adapt while (5) continuing to produce benefits for individuals/systems [34]. |
| Lean | There are two defining characteristics of Lean: Lean philosophy and Lean activities.  **Lean philosophy** is a set of core ideas that make up Lean. This is made up of two components: a commitment to Lean principles and a commitment to continuous improvement  **Lean principles** refer to an overarching set of principles aimed at transforming workplace culture. These include a focus on: eliminating waste; improving the flow of patients, providers and supplies; and ensuring all processes add value to customers. Further, Lean principles suggest that problems are identified and addressed by front line staff members as it is believed that the people doing the work are best suited to create solutions.  **Commitment to continuous improvement** refers to the acknowledgement that Lean doesn’t occur as a single intervention but instead requires a dedication to continually improving the workplace.  **Lean activities** are a set of management practices, tools, or techniques that can be directly observed and are prescribed to improve the workplace. There are two types of Lean activities: assessment activities and improvement activities.  **Lean assessment activities** work as analytic tools to identify waste and areas of possible improvement. These activities allow team members to see problems and identify opportunities to reduce waste and make improvements, but do not prescribe specific solutions. Lean assessment activities include Value Stream Mapping (VSM); spaghetti diagrams; RPIWs; Gemba walks; and root cause analysis.  **Lean improvement activities** suggest specific ways to reduce waste and improve the workplace and set up new working practices. These include actions and concepts such as: 5S events; Levelled production; DVM (including Kanban supply management); Standard Work; and Stop the Line techniques [18]. |
| Normalization Process Theory (NPT) & sense-making | A middle range theory with four key constructs (coherence, cognitive participation, collective action and reflexive monitoring) that offers potential mechanisms that promote or inhibit the embedding of complex interventions into routine everyday practice and the likelihood of sustainability [30, 31].  Four constructs: 1) **Coherence,** the **sense-making work** that people do individually and collectively when they are faced with the problem of operationalizing some set of practices, 2) **Cognitive Participation,** the **relational work** of what people do to build and sustain a community of practice around a new technology or complex intervention, 3) **Collective Action,** the **operational work** that people do to enact a set of practices, whether these represent a new technology or complex healthcare intervention and, 4) **Reflexive Monitoring,**  the **appraisal work** that people do to assess and understand the ways that a new set of practices affect them and others around them [31].  Sense-making is the process through which people assign meaning to experience [72, pg 13], effective sense-making is more likely to result in productive action. People act based on the sense they have made of something (e.g. Lean implementation). |
| NHS Sustainability Model | The NHS SM model provides ten contextual factors that potentially explain and increase the likelihood of sustainability and continuous improvement. The factors are grouped into three domains: process, staff and organization [32].  **Process factors** (n=4)  Benefits  Credibility of evidence  Adaptability  Monitoring progress  **Staff Factors** (n=4)  Training and involvement  Attitudes  Senior leaders  Clinical leaders  **Organization Factors** (n=2)  Infrastructure  Fits with goals and culture |
| Implementation | Implementation is an actively planned and deliberately initiated effort with the intention to bring a given intervention into policy and practice within a particular setting. These actions are undertaken by agents who either actively promote the use of the intervention or adopt the newly appraised practices. Usually, a structured implementation process consisting of specific implementation strategies is used and underpinned by an implementation theory. The implementation process is an active, multistage, iterative and dynamic process that does not usually occur in a linear fashion [70, pg 6]. |
| Setting & system levels | Four levels of change in health system: the individual (micro level), the group or team, the organization (meso level) and the larger system or environment (macro level) in which individual organizations are embedded [71, pg 283].  Setting refers to the specific physical location, in which the intervention is put into practice and interacts with context and implementation [70, pg 6]. |
| System, process & clinical problems | Clinical: a**:**involving direct observation of the patient clinical diagnosis, b**:**based on or characterized by observable and diagnosable symptoms clinical treatment [73].  Process: A series of actions or steps (procedures) taken in order to achieve a particular end (outcome) [74].  System: a: a set of detailed methods, procedures and routines created to carry out a specific activity, perform a duty, or solve a problem, b: an organized, purposeful structure that consists of interrelated and interdependent elements (components, entities, factors, members, parts etc.). These elements continually influence one another (directly or indirectly) to maintain their activity and the existence of the system, in order to achieve the goal of the system [74]. |
