# Supplementary material for: The sustainability of Lean in pediatric healthcare: a realist review
Source: Syst Rev. 2018 Sep 11;7:137. doi: 10.1186/s13643-018-0800-z (PMC6134523; doi:10.1186/s13643-018-0800-z)
Supplement: Supplementary file 2 — CINAHL search strategy. Search applied for the review. (DOCX 32 kb) [file 13643_2018_800_MOESM2_ESM.docx]

## Additional file 2 Review search strategy

|  | **Database: CINAHL via EbscoHost**  **Search Date: June 8, 2016** |  |
| --- | --- | --- |
| **#** | **Query** | **Results** |
| 1 | ( TI (lean and (admitting or clinic or clinics or emergency department? or emergency medicine or emergency room? or emergency service? or family practice? or general practice? or healthcare or health care or hospital? or hospitali#ed or inpatient? or intensive care or ICU or "length of stay or nursing" or oncology or outpatient? or patient care or pharmacist? or pharmacy or physician? or practitioner? or primary care or readmission? or surgeon? or surgery or surgical or trauma center? or trauma centre? or trauma service? or trauma care or ward or wards)) ) OR ( AB (lean W4 (admitting or clinic or clinics or emergency department? or emergency medicine or emergency room? or emergency service? or family practice? or general practice? or healthcare or health care or hospital? or hospitali#ed or inpatient? or intensive care or ICU or "length of stay or nursing" or oncology or outpatient? or patient care or pharmacist? or pharmacy or physician? or practitioner? or primary care or readmission? or surgeon? or surgery or surgical or trauma center? or trauma centre? or trauma service? or trauma care or ward or wards)) ) | 147 |
| 2 | ( TI (lean and (approach or business model? or care or collaborat* or design* or enterpri?e or healthcare or health care or implementation? or industry or initiative? or intervention* or leader* or management or methodolog* or method? or organi?ation* or plan or planning or philosophy or practice or practices or principles or principle or process improvement? or production or program? or programme or programmes or quality or redesign* or reengineer* or restructur* or reorgani* or safety or sigma or strategy or strategies or thinking or tool or tools or workshop*)) ) OR ( AB (lean W3 (approach or business model? or care or collaborat* or design* or enterpri?e or healthcare or health care or implementation? or industry or initiative? or intervention* or leader* or management or methodolog* or method? or organi?ation* or plan or planning or philosophy or practice or practices or principles or principle or process improvement? or production or program? or programme or programmes or quality or redesign* or reengineer* or restructur* or reorgani* or safety or sigma or strategy or strategies or thinking or tool or tools or workshop*)) ) OR ( TI think lean OR AB think lean ) | 500 |
| 3 | ( TI (lean W3 (workflow? or efficienc* or efficient*)) ) OR ( AB (lean W3 (workflow? or efficienc* or efficient*)) ) OR ( TI (Lean and waste) or AB (lean W3 waste) ) | 21 |
| 4 | ( TI (lean technique? or lean manufacturing) ) OR ( AB (lean technique? or lean manufacturing) ) OR ( TI (lean basics or lean training) OR AB (lean basics or lean training) ) | 551 |
| 5 | ( TI (fishbone and (ishikawa? or diagram? or analys* or chart? or lean)) ) OR ( AB (fishbone W2 (ishikawa? or diagram? or analys* or chart? or lean)) ) OR ( TI pareto diagram* OR AB pareto diagram* ) | 17 |
| 6 | ( TI (quality lost function* or quality function deploy*) ) OR ( AB (quality lost function* or quality function deploy*) ) OR ( TI (5S and (lean or production or industr* or manufactur* or quality improv* or management or CQI)) ) | 209 |
| 7 | ( AB (5S W5 (lean or production or industr* or manufactur* or quality improv* or management or CQI)) ) OR ( TI (5S W15 (sort or shine or sweep or straighten or standardi*)) ) OR ( AB (5S W15 (sort or shine or sweep or straighten or standardi*)) ) | 5 |
| 8 | ( TI (5s W3 (event? or method? or methodolog* or model? or process or processes or safety or waste or quality)) ) OR ( AB (5s W3 (event? or method? or methodolog* or model? or process or processes or safety or waste or quality)) ) OR ( TI takt time OR AB TAKT TIME ) | 8 |
| 9 | ( TI kanban OR TI (poka yoke? or poka yok?) OR AB (poka yoke? or poka yok?) OR TI DMAIC OR AB DMAIC ) OR ( TI (Define W2 Measure W2 Analy?e W2 Improve W2 Control) OR AB (Define W2 Measure W2 Analy?e W2 Improve W2 Control) ) OR ( TI total productive maintenance OR AB total productive maintenance ) | 27 |
| 10 | ( TI single minute exchange? OR AB SINGLE MINUTE EXCHANGE OR TI jidoka OR AB jidoka ) OR ( TI value stream map* OR AB value stream diagram* ) OR ( TI (7 wastes or seven wastes or (7S W5 (waste? or lean or management or production or organi?ation))) ) | 32 |
| 11 | AB (7 wastes or seven wastes or (7S W5 (waste? or lean or management or production or organi?ation))) | 87 |
| 12 | ( TI (7S and (quality improv* or cqi or total quality or lean or production or industr* or manufactur* or quality improv* or management or CQI)) OR AB (7S W10 (quality improv* or cqi or total quality or lean or production or industr* or manufactur* or quality improv* or management or CQI)) ) OR ( TI ((total quality or quality assurance or quality improvement) and lean) OR AB ((total quality or quality assurance or quality improvement) W10 lean) ) AND ( TI (standardi?ed work or standardi?ed workflow?) OR AB (standardi?ed work or standardi?ed workflow?) OR TI standard work OR AB STANDARD WORK ) | 6 |
| 13 | ( AB (((PDSA or PDCA or TQIS) W3 (cycle or process or processes or intervention or quality or lean or improv*)) or ("plan do study" or "plan do check")) ) OR ( TI (((PDSA or PDCA or TQIS) AND (cycle or process or processes or intervention or quality or lean or improv*)) or ("plan do study" or "plan do check")) ) | 263 |
| 14 | ( TI ((wait* time? or reduc* wait*) and lean) or AB ((wait* time? or reduc* wait*) W4 lean) ) | 11 |
| 15 | ( TI (lean and (overcrowd* or patient* flow?)) or AB (lean W4 (overcrowd* or patient* flow?)) ) | 2 |
| 16 | TI ( TOYOTA OR GEMBA OR KAIZEN ) OR AB ( TOYOTA OR GEMBA OR KAIZEN ) | 104 |
| 17 | TI (innovation? W2 collaborat*) OR AB (innovation? W2 collaborat*) | 15 |
| 18 | TI ( (PROCESS MAP? or process mapping) ) OR AB ( (PROCESS MAP? or process mapping) ) | 895 |
| 19 | TI ( (mistake proofing or value-stream map? or incident learning) ) OR AB ( (mistake proofing or value-stream map? or incident learning) ) | 382 |
| 20 | TI ( ((shewhart or shewart or deming) W3 (cycle or method*)) ) OR AB ( ((shewhart or shewart or deming) W3 (cycle or method*)) ) | 16 |
| 21 | TI process failure mode OR AB "process failure mode" | 6 |
| 22 | TI (failure mode? W2 Effect? analys*) OR AB (failure mode? W2 Effect? analys*) | 80 |
| 23 | TI FMEA OR AB FMEA | 82 |
| 24 | TI ( (breakthrough W3 (series or project or collaborative?)) ) OR AB ( (breakthrough W3 (series or project or collaborative?)) ) | 57 |
| 25 | TI rapid process improvement? OR AB rapid process improvement? | 78 |
| 26 | TI ( (rapid cycle W3 (improvement or quality or process or processes)) ) OR AB ( (rapid cycle W3 (improvement or quality or process or processes)) ) | 47 |
| 27 | TI quality improvement? tool? OR AB quality improvement? tool? | 189 |
| 28 | TI ( (virginia mason or releasing time to care or productive ward) ) OR AB ( (virginia mason or releasing time to care or productive ward) ) | 158 |
| 29 | TI LEAN MANAGEMENT | 47 |
| 30 | ( (MH "Quality of Health Care+") OR (MH "Quality Management, Organizational") OR (MH "Quality Improvement+") OR (MH "Quality Assessment+") OR (MH "Quality of Nursing Care") OR (MH "Quality Circles") OR (MH "Quality Assurance+") ) AND TI LEAN | 285 |
| 31 | S1 OR S2 OR S3 OR S4 OR S5 OR S6 OR S7 OR S8 OR S9 OR S10 OR S11 OR S12 OR S13 OR S14 OR S15 OR S16 OR S17 OR S18 OR S19 OR S20 OR S21 OR S22 OR S23 OR S24 OR S25 OR S26 OR S27 OR S28 OR S29 OR S30 | 3,638 |
| 32 | (MH "HOSPITALS, PEDIATRIC") OR (MH "Pediatric Physical Therapy") OR (MH "Pediatric Units+") OR (MH "Intensive Care Units, Pediatric+") OR (MH "Pediatric Occupational Therapy") OR (MH "Society of Pediatric Nurses") OR (MH "Pediatric Oncology Nursing") OR (MH "National Association of Pediatric Nurse Associates and Practitioners") OR (MH "Association of Pediatric Oncology Nurses") OR (MH "Childhood Neoplasms") OR (MH "Rehabilitation, Pediatric") OR (MH "Pediatric Cardiology") OR (MH "Pediatric Surgery") OR (MH "Pediatric Nurse Practitioners") | 23,771 |
| 33 | (MH "Pediatricians") OR (MH "American Academy of Pediatrics") | 2,303 |
| 34 | (MH "Intensive Care Units, Neonatal") OR (MH "Neonatal Assessment+") OR (MH "Neonatal Intensive Care Nursing") OR (MH "Intensive Care, Neonatal+") OR (MH "Congenital, Hereditary, and Neonatal Diseases and Abnormalities+") OR (MH "Anemia, Neonatal+") OR (MH "Neonatal Sepsis") OR (MH "Neonatal Nurse Practitioners") | 89,149 |
| 35 | (MH "Infant+") OR (MH "Infant, Newborn+") OR (MH "Child+") OR (MH "Infant, Low Birth Weight+") | 324,826 |
| 36 | (MH "Adolescent, Hospitalized") OR (MH "Adolescent Health Services") OR (MH "Adolescent Psychology") OR (MH "Adolescent Psychiatry") OR (MH "Adolescent Medicine") | 4,686 |
| 37 | (MH "Childhood Neoplasms") OR (MH "Early Childhood Intervention") | 5,815 |
| 38 | (MH "Perinatology") | 152 |
| 39 | (MH "Adolescence") | 229,318 |
| 40 | TI (adolescent? or child or children or juvenile? or teen? or teenager? or youth or infant? or neonat* neo-nat*) | 139,009 |
| 41 | TI ( (paediatric* OR pediatric*) ) OR AB ( (paediatric* OR pediatric*) ) | 52,339 |
| 42 | TI newborn? OR AB newborn? | 5,016 |
| 43 | AB ((adolescent? or child or children or juvenile? or teen? or teenager? or youth or infant? or neonat* neo-nate? or neo-natal*) W6 (care or healthcare or health or medical or hospital? or GP or general practitioner? or nurse or nurses or nursing or family practice? or general practice or family medicine or surger* or surgeon* or specialist?)) | 24,593 |
| 44 | SO ADOLESCEN* OR PEDIATRIC* OR PAEDIATRIC* OR CHILD? OR CHILDREN? OR JUVENILE? OR CHILDHOOD | 88,001 |
| 45 | MW CHILD OR CHILDREN OR PEDIATRIC* OR PAEDIATRIC* | 301,207 |
| 46 | S32 OR S33 OR S34 OR S35 OR S36 OR S37 OR S38 OR S39 OR S40 OR S41 OR S42 OR S43 OR S44 OR S45 | 565,279 |
| 47 | S31 AND S46 | 495 |
